# Silica-immobilized ionic liquid Brønsted acids as highly effective heterogeneous catalysts for the isomerization of *n*-heptane and *n*-octane†

**DOI:** 10.1039/d0ra00556h

**Published:** 2020-04-17

**Authors:** Abhishek Dhar, Nadavala Siva Kumar, Mehul Khimani, Ahmed S. Al-Fatesh, Ahmed A. Ibrahim, Anis H. Fakeeha, Hiren Patel, Rohit L. Vekariya

**Affiliations:** Department of Chemistry, Modern Institute of Engineering and Technology Bandel Hooghly 712123 West Bengal India; Department of Chemical Engineering, King Saud University P.O. Box 800 Riyadh 11421 Saudi Arabia; School of Sciences, P P Savani University NH-8, GETCO, Near Biltech, Village: Dhamdod, Kosamba, Dist Surat 394125 Gujarat India; Department for Management of Science and Technology Development, Ton Duc Thang University Ho Chi Minh City Vietnam rohit.vekariya@tdtu.edu.vn; Faculty of Applied Sciences, Ton Duc Thang University Ho Chi Minh City Vietnam

## Abstract

Metal-free imidazolium-based ionic liquid (IL) Brønsted acids 1-methyl imidazolium hydrogen sulphate [HMIM]HSO_4_ and 1-methyl benzimidazolium hydrogen sulphate [HMBIM]HSO_4_ were synthesized. Their physicochemical properties were investigated using spectroscopic and thermal techniques, including UV-Vis, FT-IR, ^1^H NMR, ^13^C-NMR, mass spectrometry, and TGA. The ILs were immobilized on mesoporous silica gel and characterized by FT-IR spectroscopy, scanning electron microscopy, Brunauer–Emmett–Teller analysis, ammonia temperature-programmed desorption, and thermogravimetric analysis. [HMIM]HSO_4_@silica and [HMBIM]HSO_4_@silica have been successfully applied as promising replacements for conventional catalysts for alkane isomerization reactions at room temperature. Isomerization of *n*-heptane and *n*-octane was achieved with both catalysts. In addition to promoting the isomerization of *n*-heptane and *n*-octane (a quintessential reaction for petroleum refineries), these immobilized catalysts are non-hazardous and save energy.

## Abstract 2

Metal-free imidazolium-based ionic liquid (IL) Brønsted acids 1-methyl imidazolium hydrogen sulphate [HMIM]HSO_4_ and 1-methyl benzimidazolium hydrogen sulphate [HMBIM]HSO_4_ were synthesized.

## Graphical abstract

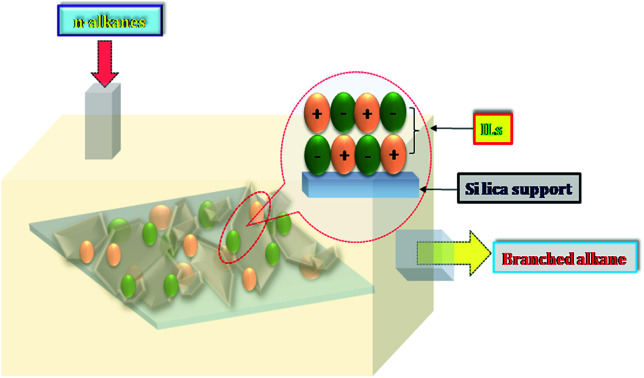

## 1. Introduction

Ionic liquids (ILs) are molten salts having melting points below 100 °C. They have received considerable attention over the last decade, due to their numerous inherent features, including low melting temperatures, stability at elevated temperatures and in air, high ionic conductivities, favorable behaviors in solution, large electrochemical windows, low vapor pressures, low volatilities, and non-flammability.^1–10^ ILs, also referred to as room-temperature ILs (RTILs), have been thoroughly investigated as alternatives to conventional catalysts^11,12^ and are prized for applications in modern green technologies.^13,14^ They are used in various organic syntheses as solvents or catalysts, such as alkylation of aromatics with olefins,^15,16^ isomerization of light alkanes,^17–19^ and oligomerization of butanes.^20–22^

Homogeneous superacid systems have been shown to catalyze alkane hydroisomerization at temperatures as low as 25 °C. These catalysts generally comprise a mixture of strong Lewis acids (*e.g.*, SbF_5_, TaF_5_, NbF_5_) and a Brønsted acid (CF_3_COOH or CF_3_SO_3_H).^23^ However, free HF in these homogeneous superacids makes them highly toxic and corrosive. The two major obstacles to the application of ILs remain cost and availability. Although a wide range of IL synthetic approaches have been published, most of these methods are complex and require extensive purification after synthesis in order to afford pure, dry RTILs. In addition, the high cost of some ILs limits their commercial availability and application. Thus, isomerization by ILs remains a topic of considerable interest worldwide.^24–26^

Global investigations have led to the concept of immobilized ILs.^14,18^ For example, ILs have been anchored to the exterior facets and cavities of various porous solid materials to make ILs more cost-efficient. The thin IL layer allows for faster diffusion and mass transfer, due to the higher relative viscosities of many RTILs. Catalysts are physisorbed on silica gel to improve IL yields, simplify purification, lengthen catalyst longevity, reduce exposure to hazardous chemicals, and increase recyclability. Applications of such catalysts are a worldwide priority.

Acids having a silica gel matrix, for example silicate polyphosphoric acid and silicate HClO_4_, have been investigated for organic fabrication due to their inherent characteristics, such as high performance, high thermal stability and recyclability, low toxicity, increased selectivity, and convenience.^27–29^ In recent reports, fluoroboric acid (a weak protonic acid) was uptake on silica to prevent unwanted side reactions.^30^ Silicate sulfuric acid has been studied as a catalyst in two different ways: as silica-adsorbed sulfuric acid and as silica sulfuric acid. The fabrication of silica-adsorbed sulfuric acid is fairly simple and inexpensive for huge scale production, since it can be simply reused without altering the activity of the catalytic system. It is therefore considered an environmentally friendly and reusable catalyst.^31^ Silica-adsorbed sulfuric acid is a strong alternative to sulfuric acid or chlorosulfonic acid, despite some limitations, for instance the loss of acidophilic functional groups, employment of toxic solvents, and requirement of costly reagents or solvents.^32^ At the same time, silica-sulfuric acid is an alternative catalyst for some specific chemical reactions to increase yield and improve other factors. In addition to silica, other solid support media have been used and numerous reports of polymer matrixes have been published. However, silica remains the preferred catalyst support.

**Scheme 1:**
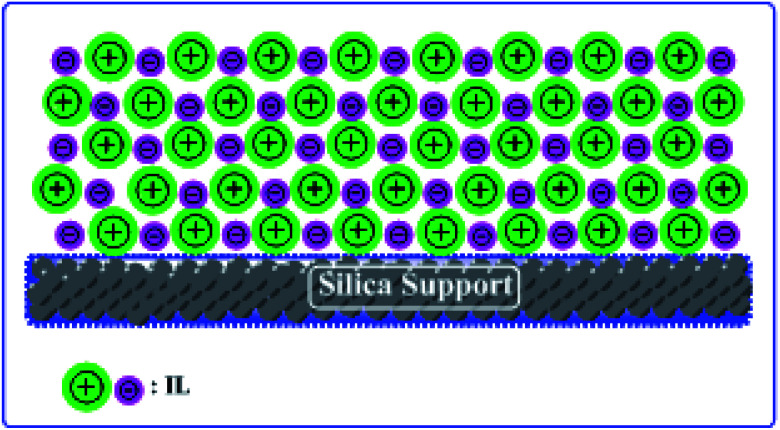
Schematic illustration of supported IL catalyst.

Herein, we report two different ILs and silica-supported catalysts. We have chosen silica gel as a support for the ILs and have prepared heterogeneous catalysts with good acidity and thermal stability (Scheme 1).

## 2. Materials

### 2.1 Chemicals

*N*-Methylimidazole, *N*-methylbenzimidazole, and silica gel Davisil® grade 633 were attained from Sigma-Aldrich. HCl, H_2_SO_4_ and other AR grade reagents were purchased and used without additional purification.

### 2.2 Methods

The vibrational modes of prepared samples were recorded in the range 4000–500 cm^−1^ with a FTIR Spectrometer (IR Prestige-21, SHIMADZU). ^1^H and ^13^C NMR spectra were registered using a NMR spectrometer (Bruker AVANCE III 400 MHz) with TMS as the internal standard and D_2_O as the solvent. TG-DTA were performed *via* a Setsys Evolution TGA-DTA/DSC, with nitrogen flow and temperature ramped at 5 °C min^−1^, from 25–800 °C. The mass spectra were recorded with “Thermo Fisher Exactive Plus High Resolution Mass Spectrometer”. Sample shapes and surface morphologies were obtained using SEM (Hitachi S-4800, Japan). Nitrogen adsorption–desorption isotherms were captured at −196 °C using a 3 Flex Micrometric (US) sorption surface unit. All samples were degassed at 130 °C for 12 h prior to reading. The total surface area has been calculated according to Brunauer–Emmett–Teller (BET) model by using software supplied with the apparatus. Total pore volumes were measured at *P*/*P*_0_ = 0.95, supposing the entire surface to be saturated with N_2_. The Barrett–Joyner–Halenda (BJH) model was used to calculate pore size distributions. Ammonia temperature-programmed desorption (NH_3_-TPD) measurements were performed on an Autochem 2920 Micrometric (US) to measure the acidity of silica-supported ILs; ammonia was used as the adsorbate. Approximately 0.2 g of sample was used in a quartz reactor and saturated with ammonia at 25 °C. Next, the samples were purged with argon to eliminate residual NH_3_ from the upperlayer of the samples. TPD was performed from 100 to 800 °C with a heating rate of 10 °C min^−1^ using argon (30 mL min^−1^) as the carrier gas.

### 2.3 Synthesis of [HMIM]HSO_4_ and [HMBIM]HSO_4_

To begin, 1-methyl imidazole (5.0 g, 0.060 mol), 1-methyl benzimidazole (5.0 g, 0.037 mol), and acetonitrile (10 mL) were mixed in a round-bottom flask with stirring for 5 min at 0 °C. Next, a fixed volume of concentrated H_2_SO_4_ was added dropwise *via* an adjusting funnel and the mixture was agitated for 1 h at 0 °C. Subsequently, the stirring was continued at 25 °C for an additional 1 h. The synthesized ILs were washed with (C_2_H_5_)_2_O to eliminate any nonionic residues and vacuum-dried at 80 °C for 24 h. Finally, viscous or oily ([HMIM]HSO_4_) and solid ([HMBIM]HSO_4_) were stored in sealed tubes under inert argon atmosphere. (Characterizations have been given in ESI: Fig. S1 and S2†).

### 2.4 UV-Vis acidity evaluation

Prior to use, ILs were dehydrated under vacuum for 2 hours at 80 °C. De-ionized water were taken in the solution preparations. UV-Vis spectra were gathered by means of spectrophotometer “Agilent B453”.

### 2.5 IL@silica preparation

**Fig. 1:**
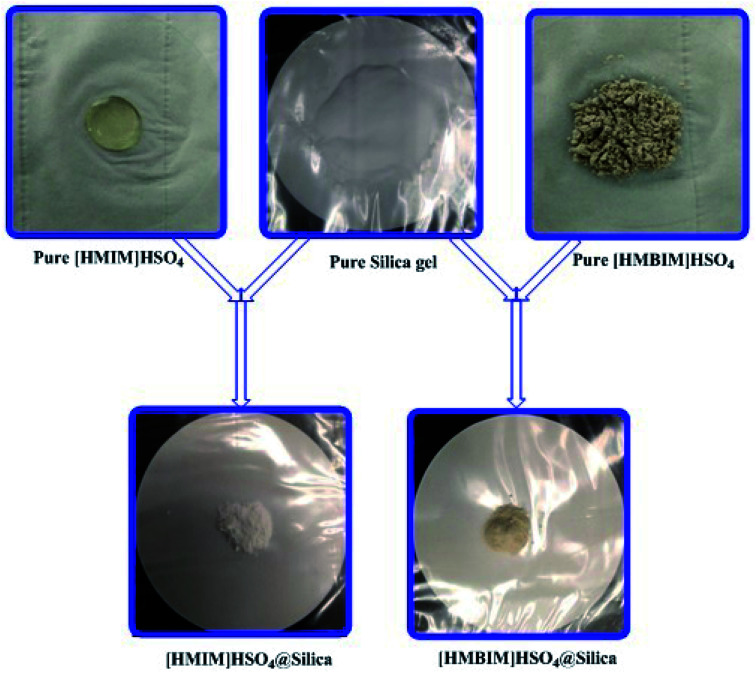
Physical appearances of silica supported IL catalysts.

Silica gel Davisil® grade 633 (average pore diameter 6 nm, pore volume 0.75 cm^3^ g^−1^, 200–425 mesh particle size) was first refluxed with 6 M HCl for 1440 min, then splashed with double distilled H_2_O to maintain the solution pH between 6 to 7, after that dried overnight at 100 °C. The combination of activated silica gel (5.0 g) and 30 mL C_2_H_5_OH was added each to two 0.1 L three-necked round-bottom flasks. Next, a solution of 4.0 g of [HMIM]HSO_4_ and a solution of 4.0 g of [HMBIM]HSO_4_ in 20 mL of C_2_H_5_OH were dropped slowly to their respective round-bottom flasks under nitrogen atmosphere. Subsequently 180 min of stirring at 25 °C, the solvent was evaporated under low pressure to get the IL@silica as a white/light brown powder (40 wt% [HMIM]HSO_4_/silica gel and [HMBIM]HSO_4_/silica gel). The physical appearances of pure ILs and ILs@silica gel are shown in Fig. 1.

### 2.6 Catalytic performance assessment

Catalytic performance was measured in a 30 mL stainless steel autoclave. A calculated amount of the feed stock (*n*-heptane and *n*-octane) and the fabricated IL catalyst were added to the autoclave, which was partially dipped in a fixed temperature oil bath, and the mixture was agitated at 1500 rpm. The pressure inside the autoclave was maintained at approximately 1 MPa and the reaction time was 12 h. Next the completion of the reaction, the autoclave was cooled to 25 °C. The reaction system separated into two phases immediately. Hence, contact time (reaction time) was considered as the time from introduction of the feedstock to cessation of stirring. The isomerized products were evaluated by gas chromatography “GC, Shanghai Haixin Chromatographic Instrument Co. Ltd., Model: GC-950FID, Column: CP-7531” after collecting liquid samples *via* syringe. The tests were performed in batch mode in the range of 20–40 °C. The IL/*n*-alkane volume ratio was fixed at optimized 0.5 : 1.^18^

## 3. Results and discussion

### 3.1 Determination of *H*_0_ values for IL Brønsted acids

UV-visible spectrophotometry was performed using an alkaline indicator to measure the Brønsted acidic strength of ILs.^33,34^ The absorbance of the non-protonated basic indicator decreases with increasing acidity of the IL. The protonated state of the indicator has a small molar absorptivity, thus [I]/[IH^+^] (where I denotes the indicator) can be calculated from the difference in absorbance measured after adding the IL Brønsted acids (ILBAs). Eqn (1) was employed to determine the Hammett function, *H*_0_. This value describes the comparative acidity of the ILs;1
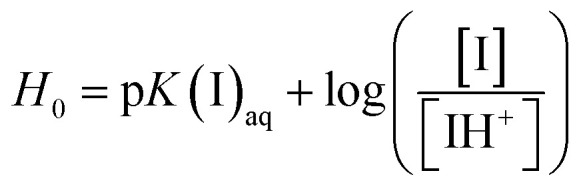


**Fig. 2:**
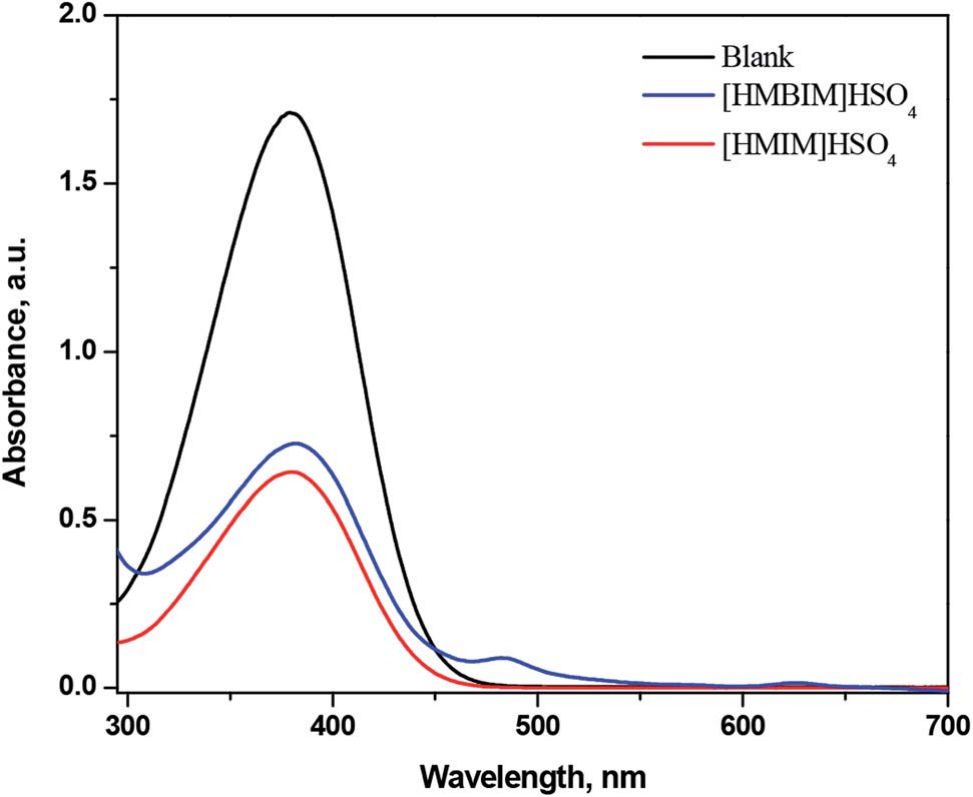
UV-Vis spectra of 4-nitroaniline after adding ILBAs in water.

Using 10 mg L^−1^ of 4-nitroaniline (p*K*(I)_aq_ = 0.99) and 25 mmol L^−1^ IL in dichloromethane, the *H*_0_ values for IL BAs were computed. The form of non-protonated indicator displayed a maximum absorbance at approximately 378 nm in water. The absorbance of the non-protonated form of the indicator diminished adding acidic IL to the solution. Fig. 2 demonstrates that the absorbance of the non-protonated form of the indicator for the two acidic ILs comes in the order: [HMBIM]HSO_4_ > [HMIM]HSO_4_.

The order of acidity of the two ILs was determined with the subsequent *H*_0_ values (Table 1): [HMIM]HSO_4_ (0.76) > [HMBIM]HSO_4_ (0.85), which confirms that [HMIM]HSO_4_ is a stronger Brønsted acid than [HMBIM]HSO_4_. It is evident from the structures of the two synthesized ILs that the [HMBIM]HSO_4_ IL provides increased steric hindrance to the acidic proton residing on N^+^, making generation of a ‘naked’ proton is less favorable. As a result, the Brønsted acidity of [HMBIM]HSO_4_ IL is reduced. The acidic strength of the ILs depends on the features of both the cations and anions. When the cations of the ILs were the same, the acidity of the IL was controlled by the anion type.

**Table 1:** Computation and assessment of *H*_0_ parameters of BAILs in H_2_O at 30 °C by using 4-nitroaniline indicator

Entry	ILs	*A*_max_	[I] (%)	[IH^+^] (%)	*H*_0_
1	No IL	1.708	100.0	0	—
2	[HMIM]HSO_4_	0.641	37.5	62.5	0.76
3	[HMBIM]HSO_4_	0.726	42.5	57.5	0.85

### 3.2 Characterization of catalysts

#### 3.2.1 FT-IR

**Fig. 3:**
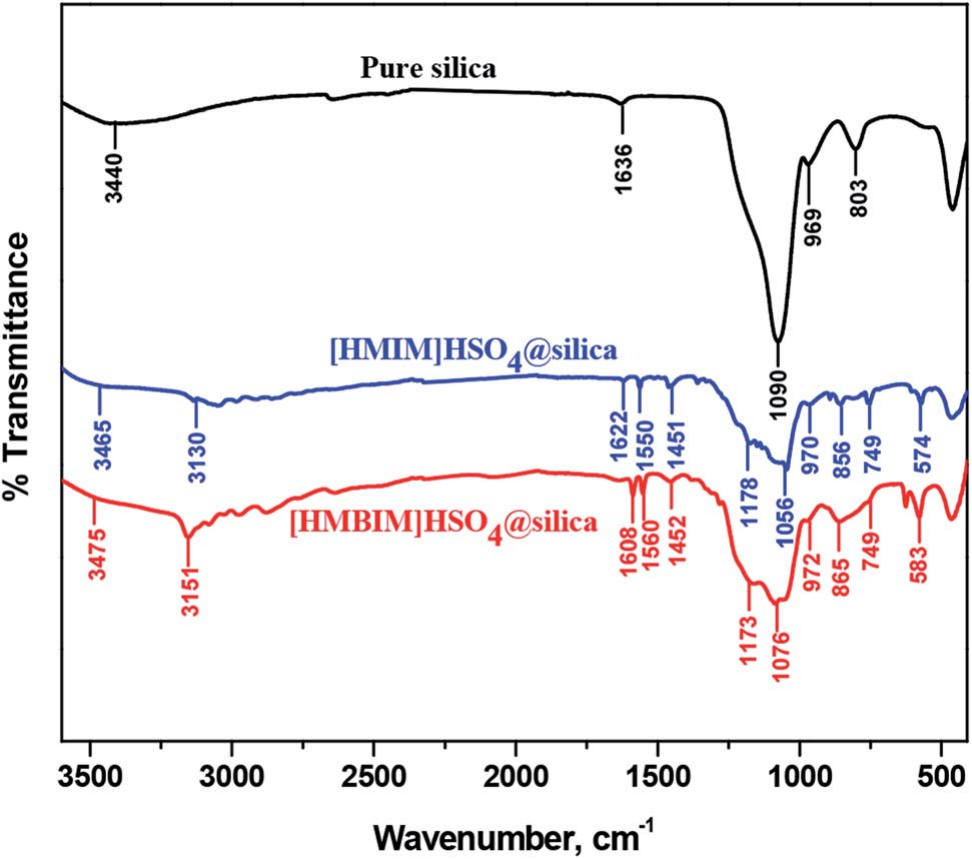
Comparison of FT-IR spectra of pure silica, [HMIM]HSO_4_@silica (CAT-1), and [HMBIM]HSO_4_@silica (CAT-2).

The synthesized catalysts were also characterized using FTIR. The spectra of silica alone and the silica-supported ILs, [HMIM]HSO_4_@silica (CAT-1) and [HMBIM]HSO_4_@silica (CAT-2), are shown in Fig. 3.

FT-IR analysis confirms the successful preparation of both the CAT-1 and CAT-2 catalysts. Characteristic spectral bands were observed for CAT-1 and CAT-2 at 749 cm^−1^ (attributed for C–H stretching), 865 and 856 cm^−1^ (C–H bending), ∼1050 cm^−1^ (S=O stretching), 1173 and 1178 cm^−1^ (C–N stretching), 1451 and 1452 cm^−1^ (C–N band of the imidazolium ring of the supported ILs). Silica support increases the effective surface area of the catalysts, which in turn enhances the isomerization reaction yields. In addition, the large covalently bonded silica network increases the thermodynamic stability of the catalysts.

#### 3.2.2 TGA

**Fig. 4:**
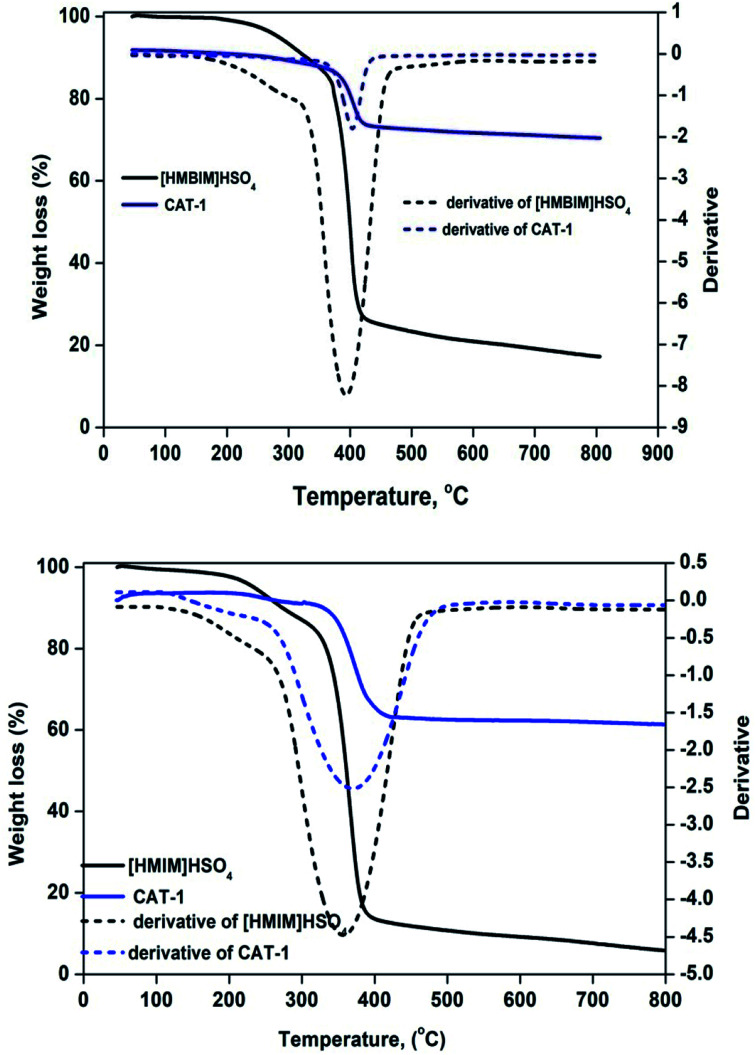
TGA curves for pure ILs and IL@silica.

ILs are considered to be thermally stable, due to their extremely high decomposition temperatures. Thermal stability measurements were performed by thermogravimetric analysis (TGA) as a function of % weight loss *vs.* temperature (°C). Fig. 4 displays TGA curves for CAT-1 and CAT-2. Decomposition temperatures (*T*_d_) can be defined by several definitions, including start, % mass loss, and peak (*T*_peak_) temperatures. Weight loss from both pure ILs and silica-supported IL catalysts at temperatures below 100 °C is ascribed to desorption of left over physisorbed H_2_O and/or organic solvent molecules from pore channels used at the beginning of catalyst preparation. The temperature at which the sample begins to lose mass is known as *T*_start_.^35^ For both ILs, *T*_start_ is approximately 110 °C. However, both supported catalysts are thermally stable up to at least 341 °C (CAT-1) and 381 °C (CAT-2). Degradation of 10% [HMIM]HSO_4_, [HMBIM]HSO_4_, CTA-1, and CTA-2 occurs at 273.1, 329.6, 359.8, and 395.4 °C, respectively.

The first derivative of the weight loss curve can be utilized to determine the point at which the most explicit weight loss occurs. The first derivative peak temperatures for [HMIM]HSO_4_, [HMBIM]HSO_4_, CTA-1, and CTA-2 are 359, 391, 365.1, and 402.5 °C, respectively. For imidazolium-based ILs, degradation occurs through a variety of mechanisms, the most common of which is loss of major alkyl chains.^36,37^ In our case, both ILs also follow this mechanism. Increasing temperature leads to cleavage of the methyl group; these results also correlate with mass spectral evidence. However, benzene substitution provides more thermal stability to the IL. The maximum degradation temperatures for [HMIM]HSO_4_ and [HMBIM]HSO_4_ are 384.7 and 413.9 °C, respectively. Beyond that temperature, no significance weight loss is observed up to 800 °C. The maximum weight losses were approximately 27.01% and 12.92% over temperature ranges of 381.2–423.5 °C and 341.7–417.7 °C for CAT-1 and CAT-2, respectively. Slow degradation at higher temperatures may attributed to the larger number of Si–O–Si bonds formed in imidazolium IL, which could hinder thermal decomposition significantly. However, at extremely high temperatures, the covalently attached ILs decomposed (dissociation of imidazolium moieties) from the top layer of the silica gel. Hence, the final observed weight losses occurred from 417.0–790.7 °C (∼2.31%) for CAT-1 and 423.5–790.0 °C (∼3.54%) for CAT-2.

#### 3.2.3 Morphological studies by scanning electron microscopy

**Fig. 5:**
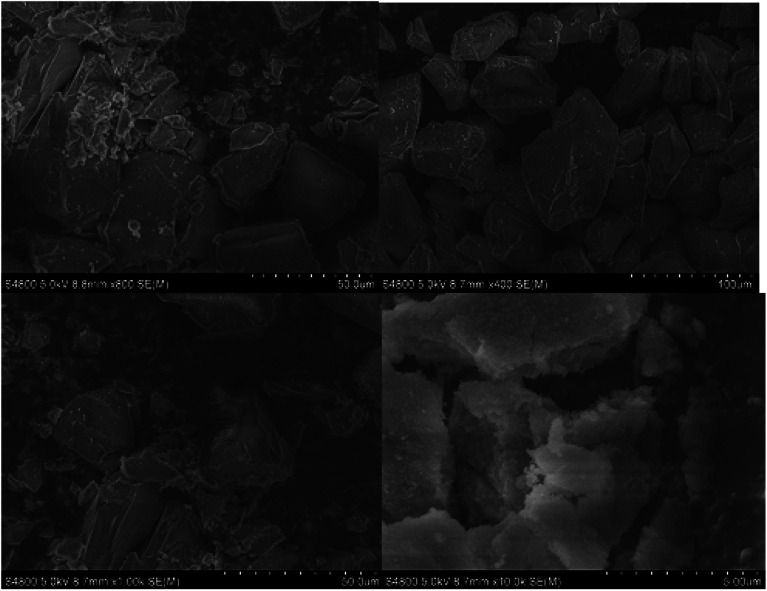
SEM images of pure silica gel (without immobilized IL).

**Fig. 6:**
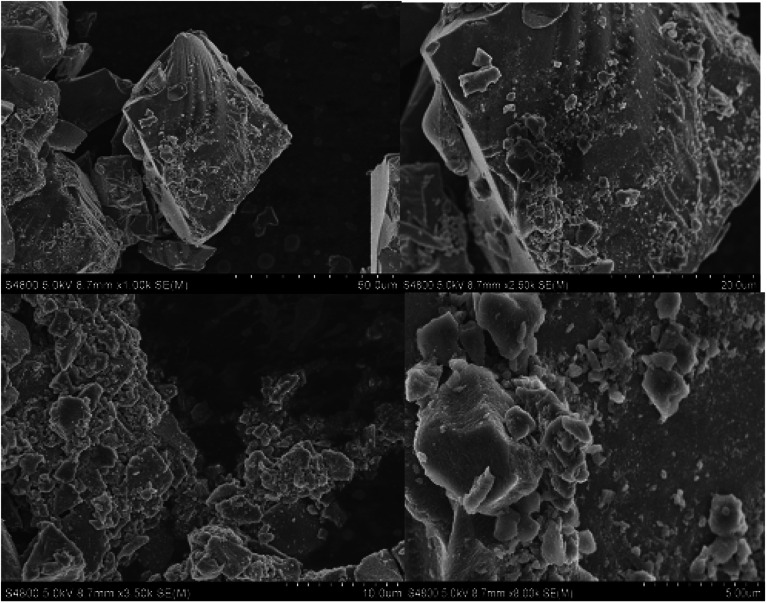
SEM images of CAT-1 (silica gel with immobilized IL (HMIM–HSO_4_)).

**Fig. 7:**
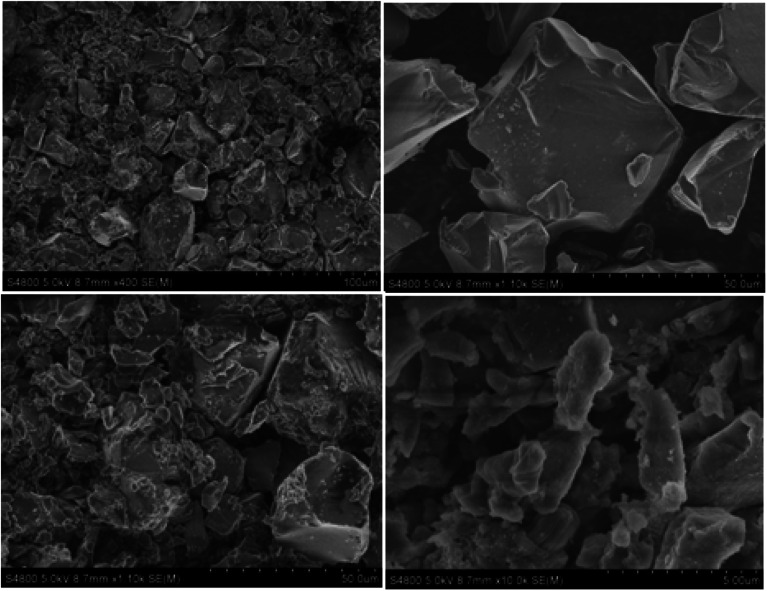
SEM images of CAT-2 (silica gel with immobilized IL (HMBIM–HSO_4_)).

SEM was used to investigate the geometry and surface properties of pure silica and silica-supported IL samples (Fig. 5–7). There is no significance difference in particle size between the silica gel and the silica-supported ILs. This indicates appreciable mechanical stability of the silica gel particles during immobilization. Nevertheless, the surface morphologies of the two samples are quite different. Fig. 5 demonstrates that the SiO_2_ surface is thin and that small agglomerates are found on the top layer of silica-supported IL (Fig. 6 and 7). No apparent changes in crystal morphology were observed when ILs were loaded onto silica at 40%, as shown in the SEM micrographs. Morphology changes are not expected to occur during sintering of the IL species, otherwise it would not be feasible to recycle the catalyst. Hence, we conclude that the silica crystals remain intact after the reversible immobilization of catalyst particle agglomerates.

#### 3.2.4 N_2_ adsorption–desorption determination of catalyst pore structures and surface areas

**Fig. 8:**
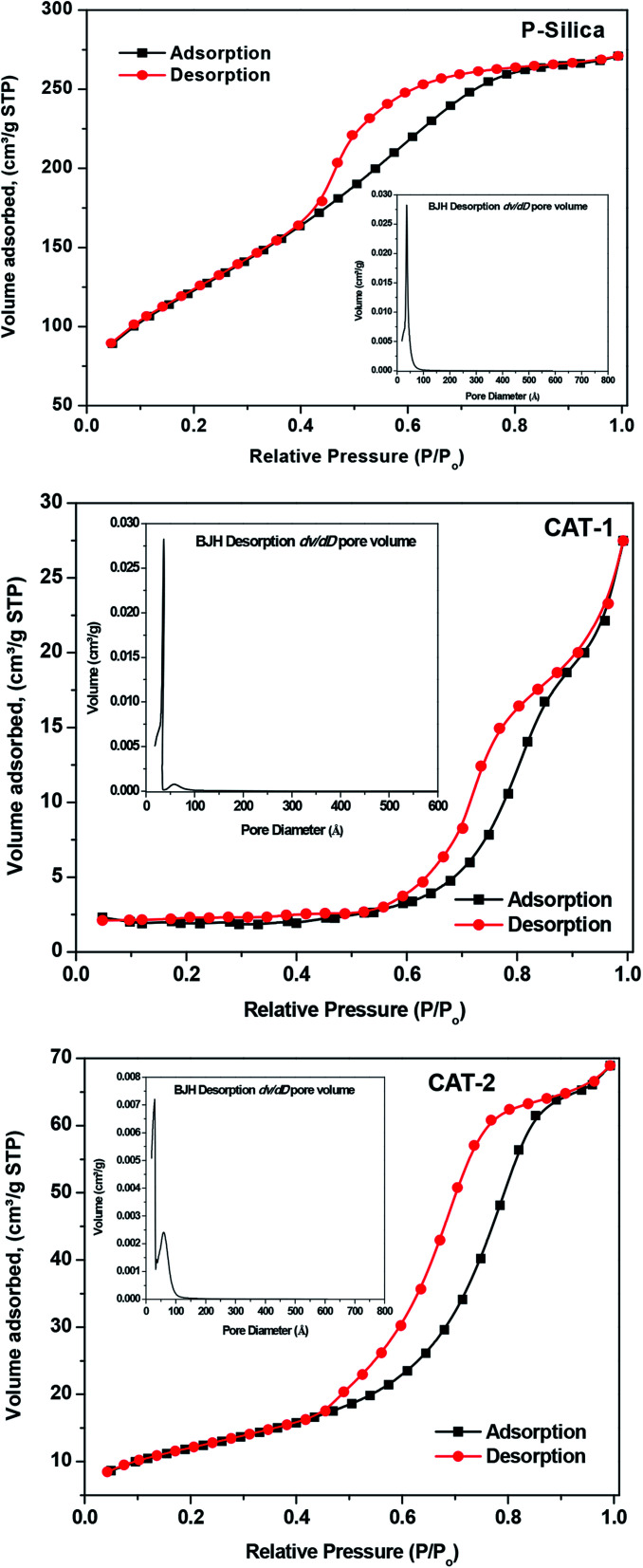
N_2_ adsorption–desorption isotherms and the corresponding BJH desorption d*V*/d*D* pore volume derived from the adsorption isotherm (inset) of pure silica and silica-supported ILs.

N_2_ adsorption–desorption experiments were used to estimate the surface aspects of CAT-1 and CAT-2 using the BET and BJH methods. The samples exhibit type-IV isotherms, confirming the mesoporous nature of the synthesized IL@silica catalysts, as well as that of silica (Fig. 8). This type of isotherm confirms capillary condensation during the adsorption process. “Ink-bottle” type pores are confirmed by the presence of H2 hysteresis loops having adsorption and desorption branches at comparative pressures of 0.8 and 0.4, respectively.^38^ Both catalysts have lower surface pore volumes and surface areas compared to pure silica gel. However, the specific surface area decreases in most cases after the introduction of the IL, due to micro- or mesopore blocking.^39^ Interestingly, comparison of the BET results for silica-supported ILs and pure silica indicates that the surface area of silica-supported ILs is less than that of pure silica. The average pore diameter and surface area of pure silica mesoporous material are 36.14 Å and 445.6 m^2^ g^−1^, respectively. Upon mixing with IL, the pore diameters increase to 85.97 Å (CAT-1) and 60.0 Å (CAT-2) and surface areas decrease to 5.45 m^2^ g^−1^ (CAT-1) and 43.14 m^2^ g^−1^ (CAT-2). Subsequently, the pore volume of 0.415 for pure silica decreases to 0.033 and 0.101 for CAT-1 and CAT-2, respectively (Table 2). The attachment of bulky imidazolium or benzimidazolium cations to the framework, which increases strain on the meso structured, is also responsible for the increased activity of the catalysts,^40,41^ since alkane molecules are more exposed at the surface, facilitating the isomerization process.

**Table 2:** Results of N_2_ adsorption measurements of the pure silica gel (P-silica) and ILs supported silica (IL@silica)

Sample	BET surface area [m^2^ g^−1^]	Single desorption pore volume [cm^3^ g^−1^]	BJH desorption average pore diameter [Å]
Pure silica gel	445.6	0.415	36.14
CAT-1 ([HMIM]HSO_4_@silica gel)	5.45	0.033	85.97
CAT-2 ([HMBIM]HSO_4_@silica gel)	43.14	0.101	60.0

Furthermore, as shown in the inset of Fig. 8, pore-size distribution was quite narrow for pure silica and widened with a new peak arising on the higher diameter side when 40 wt% IL was immobilized on silica. This indicates that ILs are perhaps confined to the silica gel poresat these concentrations.

#### 3.2.5 NH_3_-TPD acidity measurements

**Fig. 9:**
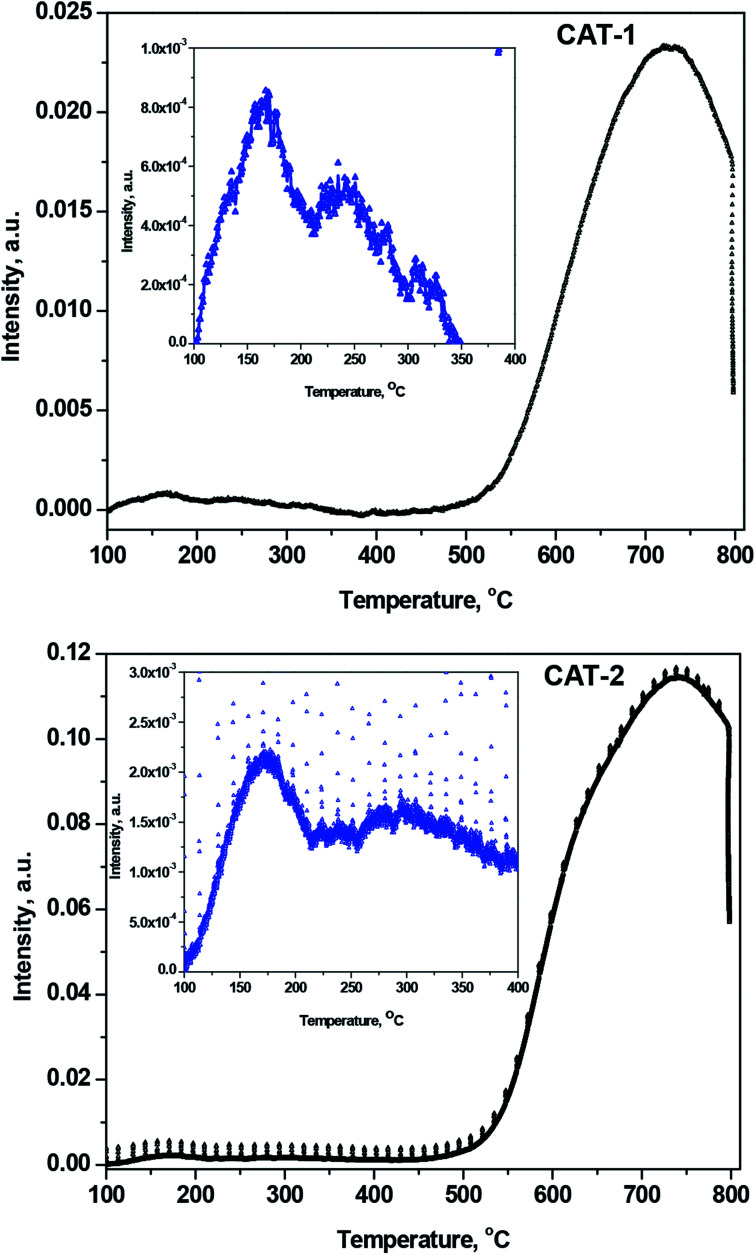
NH_3_-TPD curves for desorbed NH_3_ after heating from 100 to 800 °C for CAT-1 and CAT-2. Inset curve is the magnification of lower temperature range scale for clarity.

The band in the TPD curves can be segmented into different well-defined component bands having various maxima over the temperature range 100–800 °C (Fig. 9). The component bands indicate physisorbed, proton-held, and acid-bound NH_3_. Among the desorbed NH_3_ molecules, those appearing at ≤150 °C correspond to physically adsorbed and proton-bound NH_3_,^42,43^ while the desorbed NH_3_ molecules observed at higher temperatures correspond to acid-bound NH_3_. The amount of physisorbed and proton-bound NH_3_ desorbed at up to ∼150 °C declined with increasing temperatures, as the interlayer space of silica gel decreased after immobilization with IL.

The increased acidity of silica-supported ILs results in a greater desorption temperature for NH_3_ uptake on the acid sites.^44^ The acidity of silica-supported ILs increases due to the presence of Lewis acidic Si^4+^ centers in silica. The acid sites are defined as weak, medium, strong, and very strong, corresponding to desorption temperatures of 150–250, 250–350, 350–500, and >500 °C, respectively. Fig. 9 clearly demonstrates that only the weak, medium (inset of Fig. 9), and very strong acid sites exist in silica@IL. After heating, the number of weak acid sites decreases, and the number of medium and very strong acid sites increases.

### 3.3 Catalytic activities

Isomerization of *n*-hexane and *n*-octane in the presence of CTA-1 and CTA-2 ILs were studied with a contact time of 12 h as a function of temperature. The obtained results are displayed in Table 1. It is clear from Tables 3 and 4 that both catalysts isomerize *n*-heptane and *n*-octane to an appreciable extent. Dzhikiya *et al.*^45^ evaluated the isomerization of *n*-hexane using Pd/SO_4_/ZrO_2_/Al_2_O_3_ catalysts and determined that conversion was independent of reaction temperature. However, the yield of isomers decreased with increasing temperature. In contrast, our experimental data demonstrates that the isomerization *n*-hexane and *n*-octane to their respective isomers (2,2-dimethylpentane, 2,3-dimethylpentane, 3-ethylpentane, and 3-methyl-1-hexene) increases with temperature from 20 to 30 °C. This is due to enhanced cracking and disproportionation reactions. At high reaction temperatures, higher isomer yields are thermodynamically favorable. However, conversion of *n*-hexane decreases with a temperature increase of 20 °C, since cracking and disproportionation reactions dominate at higher temperatures. In contrast, at 40 °C, yields were approximately equal; these equilibrium yields are easily achieved at high temperatures due to high reaction rates (kinetic limitation). Ibragimov *et al.*^46^ observed similar results for the isomerization of *n*-hexane in 1-methyl-3-butylimidazolium chloride with AlCl_3_.

**Table 3:** Percentage conversion of *n*-heptane and *n*-octane to their corresponding isomers by CAT-1 after 12 h at different temperatures

Starting alkane	Name of isomers	% of isomers		
		Temperature (°C)		
		20	30	40
*n*-Heptane	2,2-Dimethyl pentane	17.1(±0.3)	19.2(±0.4)	18.9(±0.7)
	2,3-Dimethyl pentane	45.1(±1.5)	46.9(±0.1)	46.2(±0.2)
	3-Ethyl pentane	18.1(±0.1)	25.1(±0.3)	24.9(±0.4)
	3-Methyl-1-hexene	0.27(±0.01)	0.28(±0.02)	0.29(±0.02)
	*n*-Heptane	19.43(±0.3)	8.52(±0.2)	9.71(±0.3)
*n*-Octane	3-Methyl heptanes	19.1(±1.3)	21.2(±1.1)	14.8(±0.9)
	3-Ethyl hexane	23.2(±1.2)	28.6(±1.4)	21.7(±0.5)
	3,3-Dimethyl hexane	2.0(±0.04)	12.6(±0.04)	14.1(±0.1)
	2,3-Dimethyl hexane	3.1(±0.2)	18.6(±0.01)	12.4.(±1.2)
	2,3-Dimethyl-2-hexene	0.35(±0.03)	0.39(±0.04)	0.39(±0.04)
	*n*-Octane	52.25(±0.6)	37.21(±1.1)	36.61(±0.2)

**Table 4:** Percentage conversion of *n*-heptane and *n*-octane to their corresponding isomers by CAT-2 after 12 h at different temperatures

Starting alkane	Name of isomers	% of isomers		
		Temperature (°C)		
		20	30	40
*n*-Heptane	2,2-Dimethyl pentane	16.2(±0.1)	19.3(±0.2)	17.4(±0.3)
	2,3-Dimethyl pentane	42.3(±1.2)	43.1(±0.3)	42.8(±0.2)
	3-Ethyl pentane	17.1(±0.1)	24.7(±0.3)	24.3(±0.2)
	3-Methyl-1-hexene	0.25(±0.02)	0.26(±0.03)	0.25(±0.04)
	*n*-Heptane	24.15(±0.4)	12.64(±0.2)	15.25(±0.1)
*n*-Octane	3-Methyl heptanes	18.6(±0.8)	21.4(±0.8)	13.2(±0.1)
	3-Ethyl hexane	22.8(±1.1)	27.3(±1.1)	21.2(±0.3)
	3,3-Dimethyl hexane	2.1(±0.04)	10.6(±0.03)	12.4(±0.1)
	2,3-Dimethyl hexane	2.7(±0.1)	16.9(±0.03)	11.7.(±0.8)
	2,3-Dimethyl-2-hexene	0.29(±0.05)	0.33(±0.02)	0.42(±0.05)
	*n*-Octane	53.51(±0.4)	23.47(±1.1)	41.08(±0.4)

Our synthesized catalysts achieved better isomerization yields than previously reported IL catalysts, such as super acidic chloroaluminate ILs.^47^ There are several reasons for the observed increase in catalytic efficiency. Both catalysts contain Brønsted acid and Lewis acid sites, which catalyze the isomerization. In addition, incorporation of the silica matrix increases the surface area of catalyst. Importantly, the electron-withdrawing HSO_4_^−^ group makes the Si^4+^ center a stronger Lewis acid. Finally, the large bisulfate group generates electronic polarization in the material, which enhances the proton conductivity of the catalyst. Thus, Brønsted acidity increases for CAT-1 and CAT-2. A possible mechanism for the formation of the desired branched-chain isomeric alkanes follows: a ‘naked’ proton is abstracted/released from *n*-heptane or *n*-octane, resulting in the formation of a carbocation, which subsequently undergoes rearrangement to produce the isomeric alkanes. Both catalysts have isomerized *n*-heptane and *n*-octane in high yields. From Tables 3 and 4, we estimate that CAT-1 is a more effective catalyst.

## 4. Conclusions

IL Brønsted acids 1-methyl imidazolium hydrogen sulphate ([HMIM]HSO_4_) and 1-methyl benzimidazolium hydrogen sulphate ([HMBIM]HSO_4_) were synthesized and characterized by ^1^H NMR, ^13^C NMR, FT-IR, and mass spectrometry. Acidity and thermal stability of the ILs was evaluated using UV-Vis and TGA, respectively. ILs were prepared *via* a facile and inexpensive route that released no halogen by products. ILs were immobilized on silica gel to prepare silica-supported IL catalysts (SILC) [HMIM]HSO_4_@silica and [HMBIM]HSO_4_@silica. FT-IR, SEM, N_2_-adsorption–desorption (BET), NH_3_-TPD, and TGA were used to characterize the silica-supported IL catalysts. High activity and selectivity, ease of product separation, and recyclability make the immobilization of ILs catalysts advantageous. TGA data revealed that the benzene-substituted IL, [HMBIM]HSO_4_, had increased thermal stability compared to [HMIM]HSO_4_. Hence, [HMBIM]HSO_4_ is more stable. BET results demonstrated that the surface area of the silica-supported IL is smaller than that of pure silica. However, the attachment of bulky imidazolium or benzimidazolium cations to the framework contributes to higher strain on the mesostructure, which likely leads to increased catalytic activity. Both synthesized catalysts were used to isomerize *n*-heptane and *n*-octane at room temperature. Isomerization is more efficient with CAT-1 than with CAT-2. The protocol described herein is suitable for environmentally friendly industrial applications. Efforts to immobilize ILs on silica and apply them for more complex olefin and paraffin isomerization reactions are ongoing in our group.

## Conflicts of interest

No conflict of interest was stated by the authors.

## Supplementary Material

RA-010-D0RA00556H-s001
