# Supplementary material for: Silica-immobilized ionic liquid Brønsted acids as highly effective heterogeneous catalysts for the isomerization of n-heptane and n-octane
Source: RSC Adv. 2020 Apr 17;10(26):15282–92. doi: 10.1039/d0ra00556h (PMC9052301; doi:10.1039/d0ra00556h)
Supplement: RA-010-D0RA00556H-s001 [file RA-010-D0RA00556H-s001.pdf]

Electronic Supplementary Material (ESI) for RSC Advances.
This journal is © The Royal Society of Chemistry 2020

Supporting Information

Silica-immobilized ionic liquid Brönsted acids as highly effective heterogeneous catalysts for the isomerization of n-heptane and n-octane

Abhishek Dhar[§a], Nadavala Siva Kumar[§b], Mehul Khimani[§c], Ahmed S. Al-Fatesh[b], Ahmed A. Ibrahim[b], Anis H. Fakeeha[b], Hiren Patel[c], Rohit L. Vekariya[d,e*]

[a]*Department of Chemistry, Modern Institute of Engineering and Technology, Bandel, Hooghly, 712123, West Bengal, India.*

[b]*Department of Chemical Engineering, King Saud University, P.O. Box 800, Riyadh 11421, Saudi Arabia.*

[c]*School of Sciences, P P Savani University, NH-8, GETCO, Near Biltech, Village: Dhamdod, Kosamba, Dist. Surat 394125, Gujarat, India.*

[d]*Department for Management of Science and Technology Development, Ton Duc Thang University, Ho Chi Minh City, Vietnam.*

[e]*Faculty of Applied Sciences, Ton Duc Thang University, Ho Chi Minh City, Vietnam.*

2.3. Spectral data of synthesized [HMIM]HSO_4 and [HMBIM]HSO_4

[HMIM]HSO_4: *^{1}H NMR* (D_2O, δ/ppm relative to TMS) = 3H (-NCH_3), 2H (-CH aromatic protons), 1H (-NCHN); *^{13}C NMR* (D_2O δ/ppm) = 35 (-NCH_3), 119 (-CH), 121 (-CH), 135 (-NCHN); *IR* (KBr) (ν cm^{-1}) = 750 (C-H stretching), 851 (aromatic C-H bending), 1042 (S=O stretching), 1177 (C-N stretching), 1466 (C-C aromatic multiple bond stretching), 3147 (aromatic stretching), 3450 (N-H stretching). M^+ observed at m/*z* = (+) 83.06.**(Fig. S1(a-d))**

[HMBIM]HSO_4: ^{1}H NMR (D_2O, δ/ppm relative to TMS) = 3H (-NCH_3-); 2H (-CH aromatic protons), 2H (-CH aromatic protons), 1H (-NCHN); ^{13}C NMR (D_2O δ/ppm) = 32.5 (-NCH_3), 112 (-CH), 114 (-CH), 126 (-CH), 126 (-CH), 130 (-CH), 131 (-CH), 140 (-NCHN); IR (KBr) (ν cm^{-1}) = 753 (C-H stretching), 861 (aromatic C-H bending), 1038 (S=O stretching), 1177 (C-N stretching), 1461 (C-C aromatic multiple bond stretching), 3135 (aromatic stretching); M^+ observed m/*z* = (+) 133.07. **(Fig. S2(a-d))**

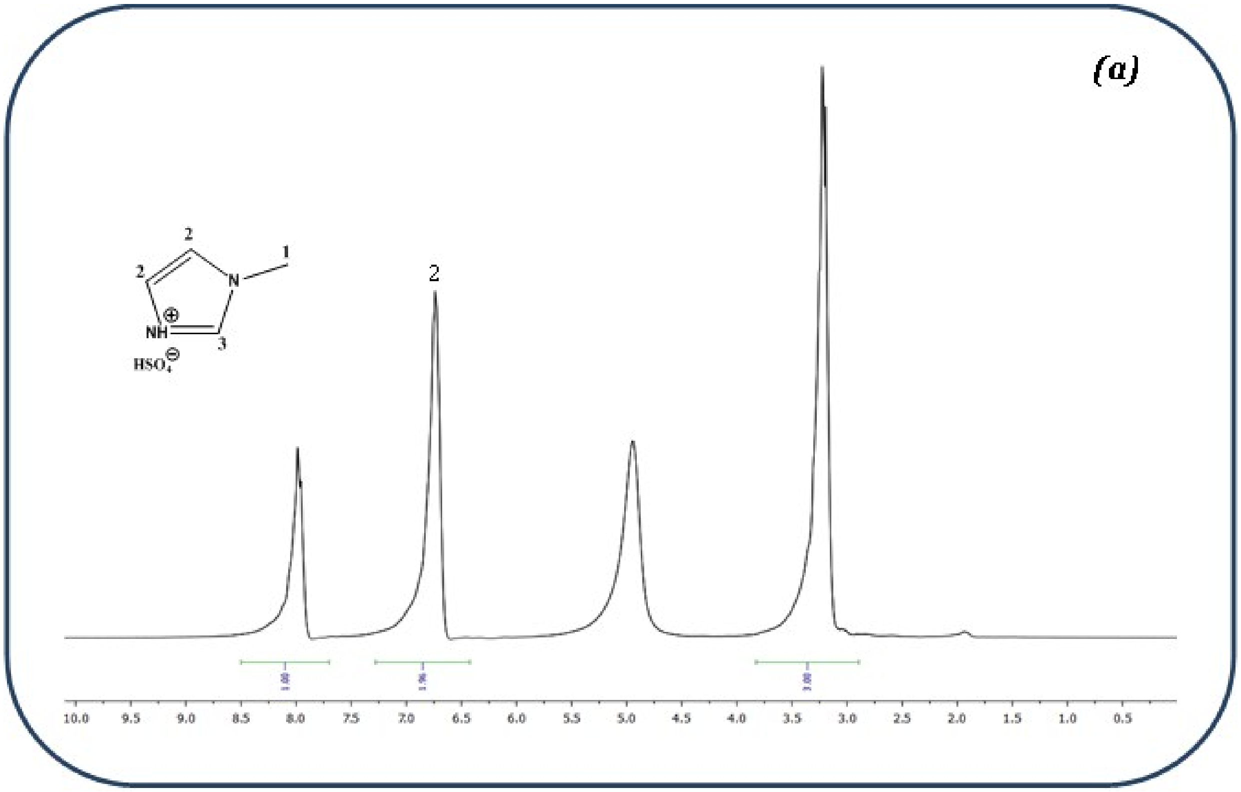
(a)
2
1
2
N
NH
3
HSO4
2
1.00
1.96
3.00
10.0 9.5 9.0 8.5 8.0 7.5 7.0 6.5 6.0 5.5 5.0 4.5 4.0 3.5 3.0 2.5 2.0 1.5 1.0 0.5

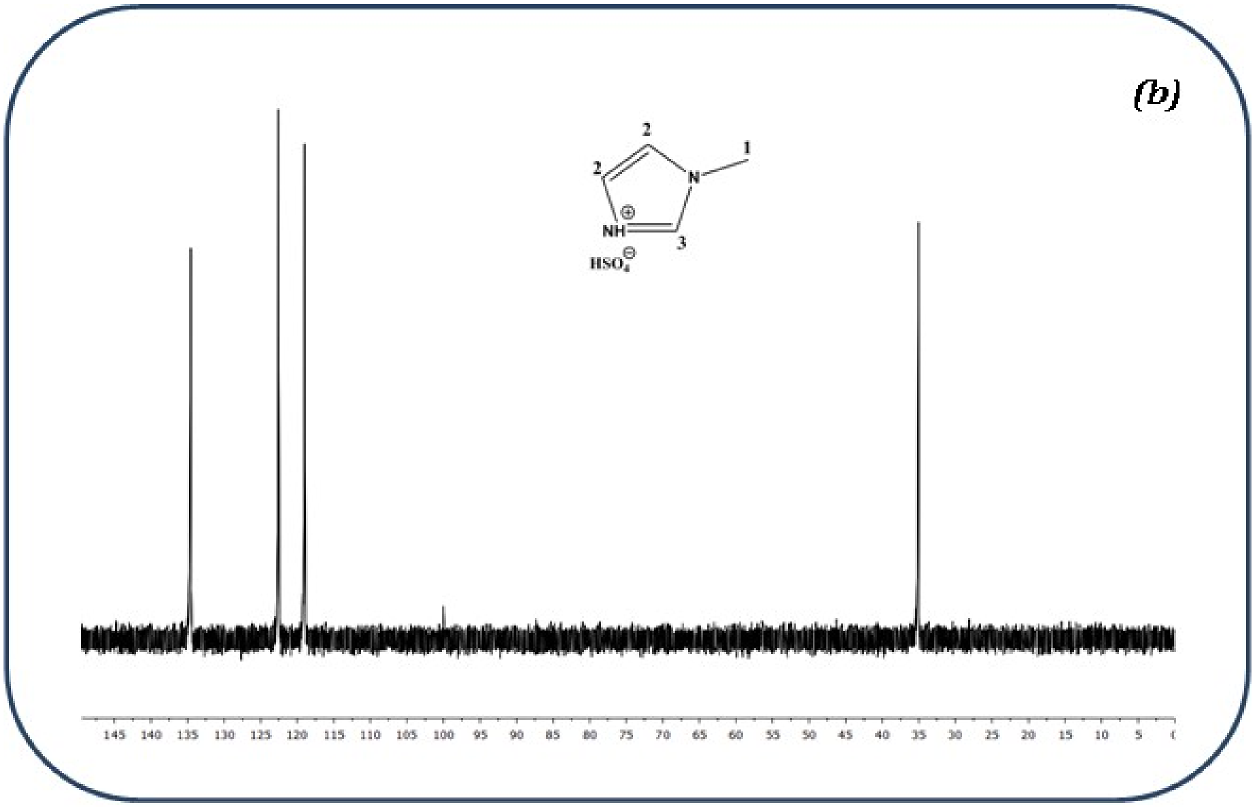
(b)
2
1
2
N
NH
3
HSO4
145 140 135 130 125 120 115 110 105 100 95 90 85 80 75 70 65 60 55 50 45 40 35 30 25 20 15 10 5

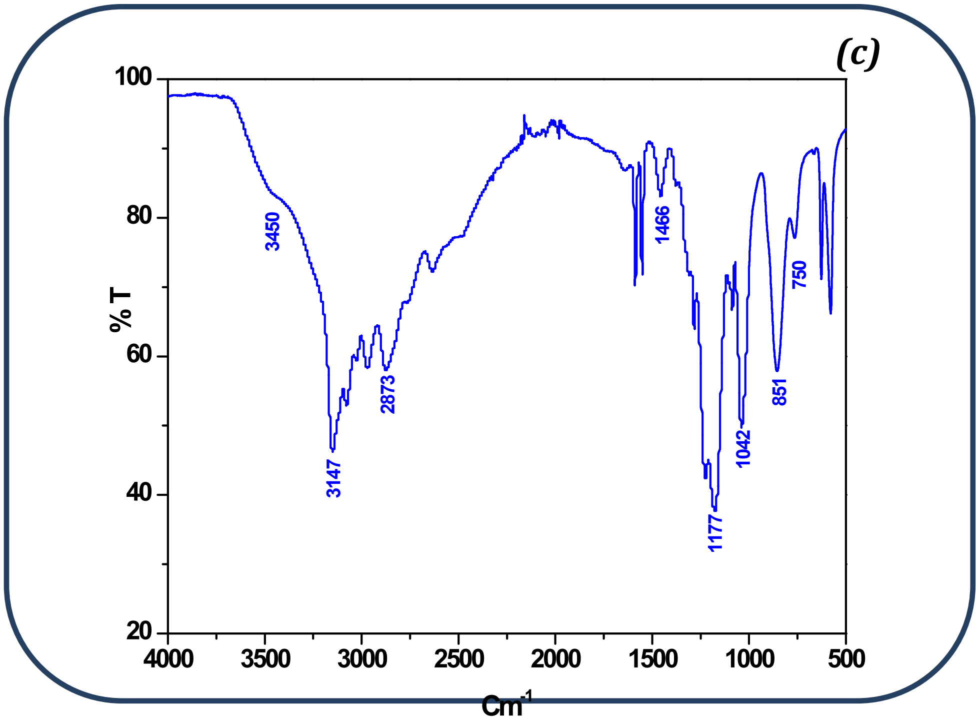

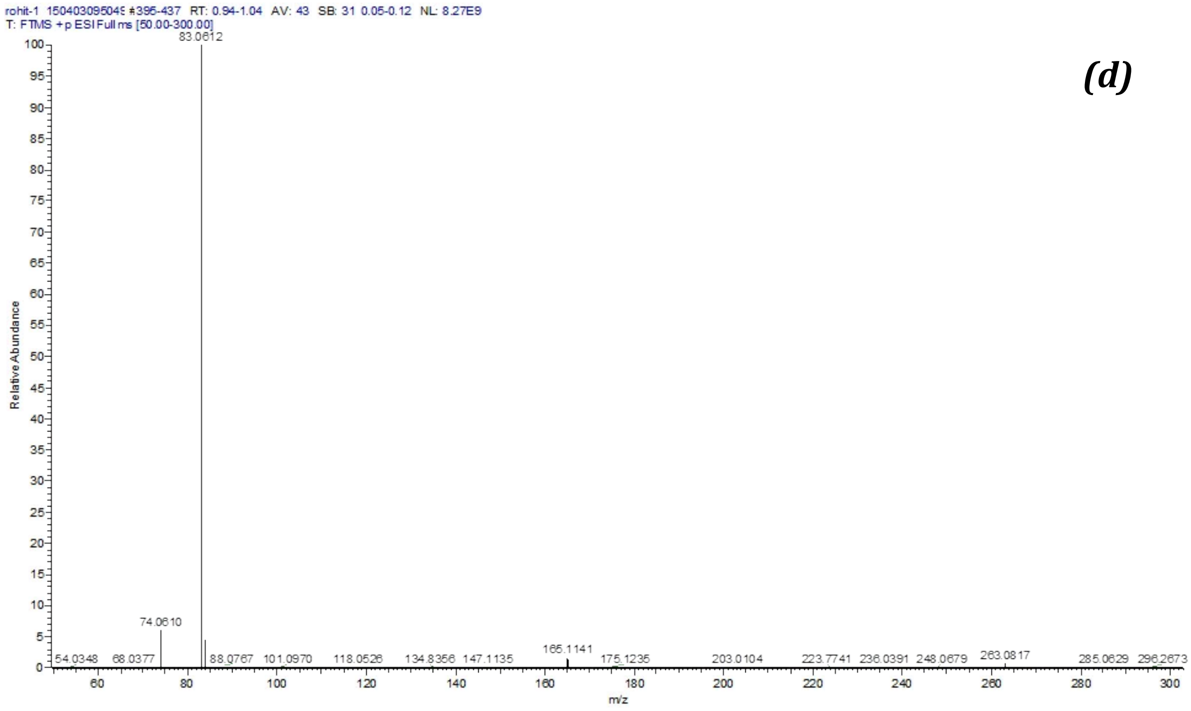

Fig.S1. (a) ^{1}H-NMR, (b) ^{13}C-NMR, (c) FT-IR and (d) Mass spectra of synthesized IL [HMIM]HSO_4.

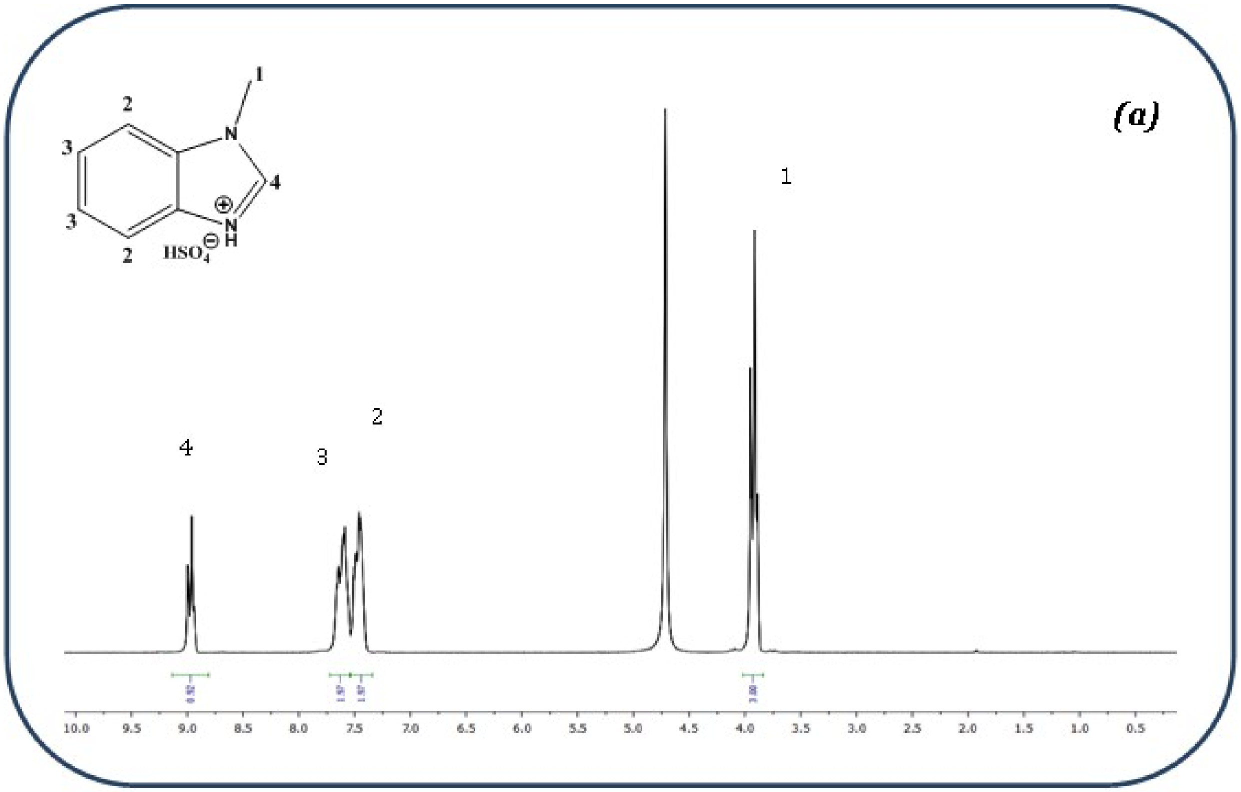
(a)
1
2
3
4
N
H
HSO4
0.92
1.97
1.97
3.00
10.0 9.5 9.0 8.5 8.0 7.5 7.0 6.5 6.0 5.5 5.0 4.5 4.0 3.5 3.0 2.5 2.0 1.5 1.0 0.5

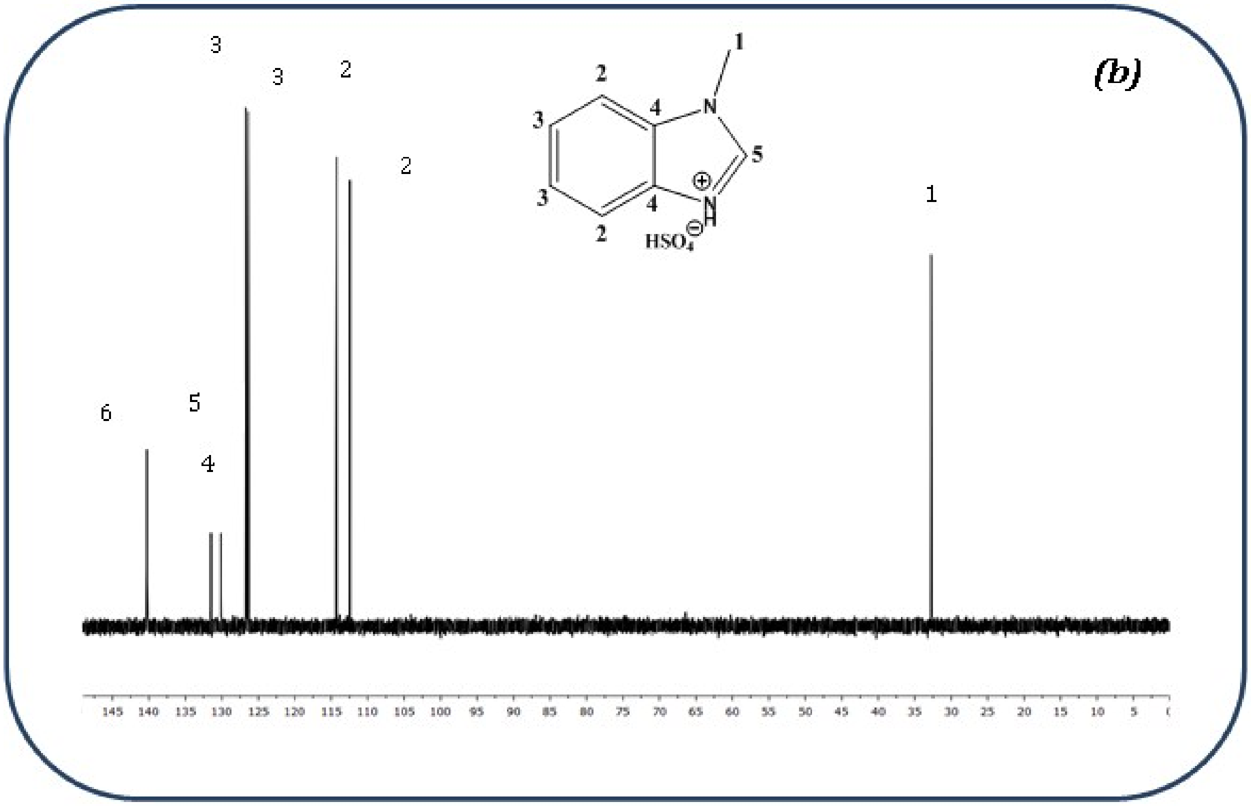
(b)
1
2
3
4
5
6
N
H
HSO4
145 140 135 130 125 120 115 110 105 100 95 90 85 80 75 70 65 60 55 50 45 40 35 30 25 20 15 10 5

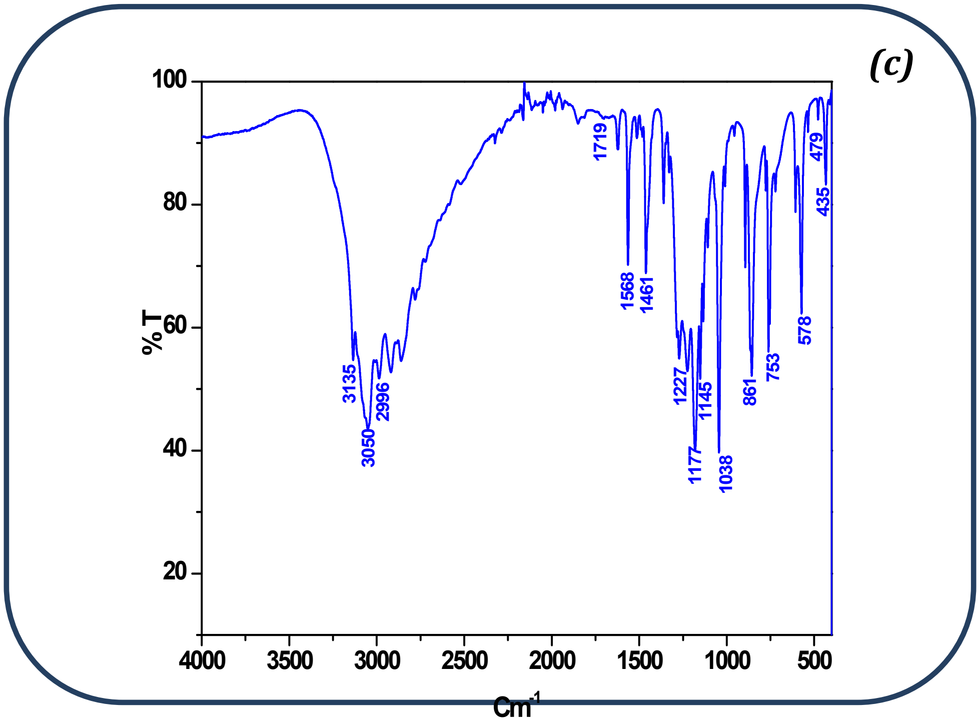

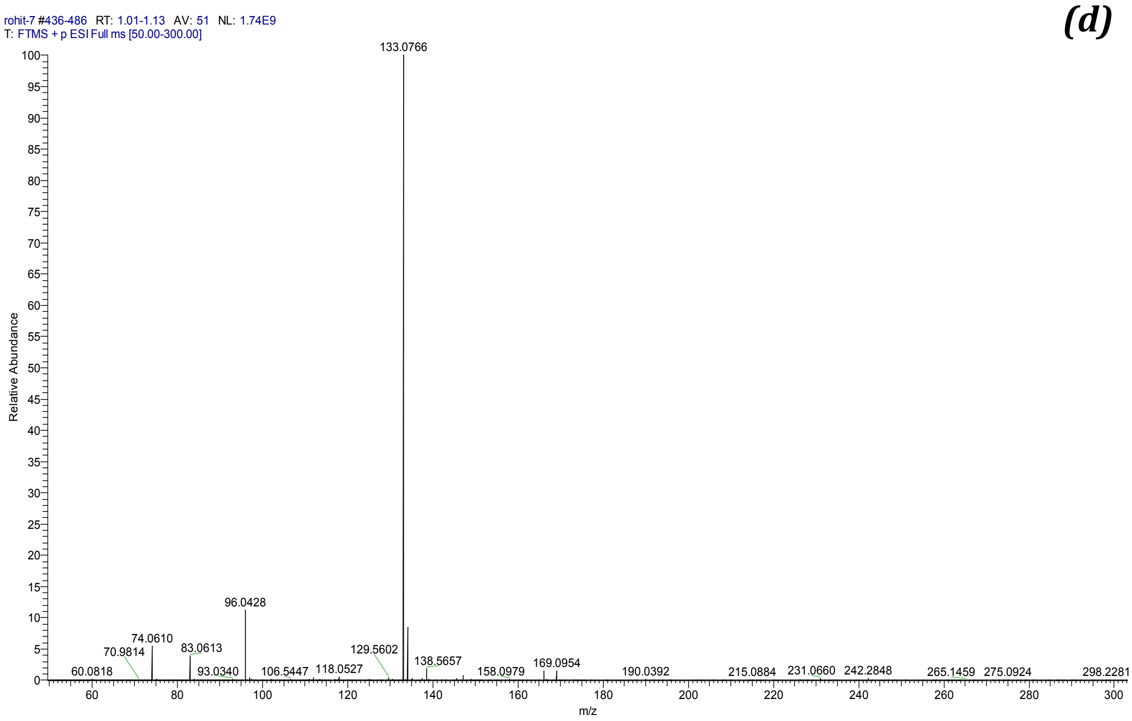

Fig. S2. (a) ^{1}H-NMR, (b) ^{13}C-NMR, (c) FT-IR and (d) Mass spectra of synthesized IL [HMBIM]HSO_4.
